# Required elements for an educational programme for lay exercise instructors in charge of community-based exercise targeting young adults with schizophrenia spectrum disorders – A stakeholder focus group study

**DOI:** 10.1186/s12888-024-05648-9

**Published:** 2024-03-26

**Authors:** Martin Færch Andersen, Kickan Roed, Victor Sørensen, Allan Riis, Bolette Skjødt Rafn, Bjørn Hylsebeck Ebdrup, Julie Midtgaard

**Affiliations:** 1 Centre for Applied Research in Mental Health Care (CARMEN), Mental Health Centre Glostrup, University of Copenhagen, Copenhagen, Denmark; 2 Department of Physiotherapy, University College of Northern Denmark, Aalborg, Denmark; 3 Center for General Practice, Aalborg University, Aalborg, Denmark; 4 Late Effects Research Center (CASTLE), Department of Oncology, Danish Cancer Society National Cancer Survivorship, Copenhagen University Hospital– Rigshospitalet, Copenhagen, Denmark; 5 Center for Neuropsychiatric Schizophrenia Research (CNSR), Mental Health Centre Glostrup, University of Copenhagen, Glostrup, Denmark; 6 Faculty of Health and Medical Science, Department of Clinical Medicine, University of Copenhagen, Copenhagen, Denmark

**Keywords:** Exercise, Schizophrenia, Personal recovery, Community-based activities, Education, Focus group, Qualitative research

## Abstract

**Background:**

Exercise plays a crucial role in addressing the increased cardiometabolic morbidity and premature mortality in people with schizophrenia spectrum disorders. When delivered in community-based settings, exercise may also reduce loneliness, while promoting overall physical activity behaviours. Skilled instructors are essential to deliver effective community-based exercise; however, knowledge about their roles and required training is lacking. We aim to explore various stakeholders’ perspectives regarding lay exercise instructors’ roles, and the required elements in an educational programme supporting the delivery of community-based exercise for young adults with SSD.

**Methods:**

We used semi-structured homogeneous focus groups with representatives from different stakeholder groups (i.e., including representatives of clinical staff within mental health, physiotherapists, exercise instructors, young adults with schizophrenia spectrum disorders, and relatives of individuals with schizophrenia spectrum disorders) targeted or affected by a community-based exercise intervention. Data were analysed using qualitative content analysis.

**Results:**

We conducted six focus groups comprising a total of 30 individuals representing five different stakeholder groups The analysis identified three categories: (i) *awareness and understanding of mental illness*, i.e., providing basic knowledge to dispel common myths and stigma regarding mental illness (ii) *protecting youth identity*, i.e., supporting the feeling of being more than just a patient, and (iii) *promoting exercise as a shared activity*, i.e., a communal pursuit, fostering personal growth among participants, their peers and the instructors.

**Conclusions:**

An educational programme for lay exercise instructors delivering community-based exercise targeting young adults with SSD should empower the instructors to assume the role of guardians of an inclusive exercise culture. Educational elements identified were adapted and integrated into an educational programme implemented and evaluated as a part of the Vega trial. Our results may be transferable to the education of lay workers in mental health care where the aim is to facilitate sustainable, recreational, community-based activities.

**Supplementary Information:**

The online version contains supplementary material available at 10.1186/s12888-024-05648-9.

## Background

People with schizophrenia spectrum disorders (SSD) face an elevated risk of developing cardiometabolic comorbidities, such as diabetes [1], metabolic syndrome, and cardiovascular disease [2], contributing to a premature mortality rate of 15–20 years [3–5]. This increased morbidity and mortality rate are partly attributed to the side effects of antipsychotic medication and unhealthy lifestyle choices, including physical inactivity and sedentary behaviour. The Lancet Psychiatry Commission [6] emphasizes that physical activity, encompassing exercise and sports, is a pivotal modifiable factor in protecting the physical health of individuals with mental illness. Moreover, increasing evidence suggests that community-based sports and exercise in groups can mitigate social isolation and reduce stigmatisation, while also enhancing the social identity of people with SSD [7–9]. Furthermore, community-based sports may offer an opportunity to deliver life skill training, improve social connectivity, and promote overall physically active behaviours within the same intervention [10]. Gym-based group exercise is both feasible [11] and popular among people with psychosis [12]. Still, people with SSD engage in less physical activity [13] and have more sedentary behaviour [14] compared to the general population. Early intervention is critical for the optimal treatment of SSD [15, 16], and the early stages of the illness may constitute a window of opportunity to establish sustainable physical activity habits when patients are younger, more active, and less affected by physical comorbidities [6, 17]. Consequently, building sustainable, easily accessible, and engaging community-based exercise and sporting activities targeting people with SSD has gained increased attention among clinicians, politicians, and stakeholders.

Qualitative studies have indicated that, for people with severe mental illness, unsupported transitioning from intention to participation is insufficient to ensure engagement in community-based exercise programs. Sustained involvement requires comprehensive practical and emotional support [9, 18], with a crucial role played by exercise instructors delivering and supervising community-based exercise programs [19]. Notably, exercise and lifestyle interventions supervised by physical educators, physiotherapists, and exercise physiologists, demonstrate greater efficacy than unsupervised interventions or those supervised by other health professionals [20]. The mental health literature recognises the value of trained lay community health workers in transitional care [21]. This emphasizes the importance of lay exercise instructors with relevant training in basic mental health literacy for promoting long-term physical activity behaviour in individuals with SSD [22]. We recently investigated the perspectives of professional experts in relation to developing community-based exercise programs for people with SSD [23]. The results of this study emphasized the importance of ensuring safe and meaningful exercise content and that exercise instructors receive formal education prior to facilitating community-based exercise for people with SSD. However, the specific elements of the role and the required skills of exercise instructors have attracted little research attention, and accordingly, the content of an educational programme remains unexplored.

Thus, the current study aims to explore various stakeholders’ perspectives regarding lay exercise instructors’ roles, and the required elements in an educational programme supporting the delivery of community-based exercise for young adults with SSD.

## Methods

### Design and setting

The study was conducted as part of the development and design of the Vega trial which aims to evaluate the effectiveness of a gym-based exercise programme delivered by non-health professional exercise instructors for young adults receiving antipsychotic medication [24]. The current study applied a descriptive qualitative design using researcher triangulation and semi-structured focus groups. The study was reported according to the Consolidated Criteria for Reporting Qualitative Research checklist [25] (Appendix 1).

### Sampling

We employed a purposeful sampling strategy to ensure information richness [26] by recruiting participants with specific knowledge, experience, and/or interest concerning the study aim. Specifically, we considered stakeholders targeted or affected by a community-based exercise intervention as relevant contributors possessing valuable knowledge about the required training of lay exercise instructors [27]. Thus, we recruited representatives from the following five main stakeholder groups: clinical staff from outpatient mental health services; physiotherapists working in mental health care; exercise instructors, both with and without previous experience with facilitating exercise for young adults with SSD; young adults with SSD; and relatives of individuals with SSD. Within the stakeholder groups, we recruited using convenience sampling through email invitations distributed by gatekeepers to the different stakeholder groups, e.g., the team leader of clinical staff, peer-board coordinator, or personal email directly to stakeholders such as exercise instructors. We aimed to conduct focus groups comprising four to seven stakeholders, as smaller groups might easier facilitate in-depth discussions [28], and used an over-recruitment strategy to account for last-minute cancellations. Considering the specific aim of the current study and examination of mutable perspectives (i.e., inclusion of various stakeholder groups and multiple representatives from each stakeholder group), we estimated that conducting a single focus group with each stakeholder group would be enough to achieve adequate information power.

### Data collection

We used semi-structured homogeneous focus groups (all focus group participants representing the same stakeholder group) which were audio recorded and facilitated by MFA (male investigator, certified physiotherapist, and full-time PhD student), who has previous experience in facilitating focus groups. VS (male investigator, human physiologist, and full-time PhD student) assisted as an observer, took notes, and posed additional questions when necessary, with a primary emphasis on evaluating density and information richness within each focus group [29]. Neither MFA nor VS had any relations with the stakeholders beforehand. The individual focus groups were held in conference rooms made available by either the stakeholders’ gatekeeper or the researchers. To facilitate group discussions, stakeholders were given three overall statements (Table 1) and instructed to rank them from most to least important, providing explanation for their chosen order. This step was followed by specific questions for individual stakeholder groups to discuss (Table 2). The overall statements were developed based on findings from our previous qualitative study examining the perspectives of clinical and professional experts on the development of community-based exercise programmes for people SSD [23]. Only the stakeholders and the researchers (MFA and VS) were present during the focus groups. The focus groups lasted ~ 90 min and were not repeated. Stakeholders received a token of appreciation of EUR 25.

**Table 1:** Overall statements to facilitate discussion during focus groups

Overall statements
*It is important that instructors learn about the treatment of schizophrenia spectrum disorders and how the disorders affect people’s lives.*
*It is important that instructors learn about the concept of recovery and how exercise can support personal recovery.*
*It is important that instructors learn about adapting the exercise content, so it is safe and relevant for people with schizophrenia spectrum disorders.*

**Table 2:** Specific questions tailored to stakeholders to facilitate discussion in focus groups

Examples of specific questions for stakeholder groups	
Clinical staff from outpatient mental health services	*What can exercise instructors do in order for community-based exercise to complement outpatient mental health services?*
Exercise instructors with/without experience in coaching people with SSD	*What concerns do you have about facilitating community-based exercise targeting young adults with schizophrenia?*
Physiotherapists working in mental health services.	*What is important to consider when working with community-based exercise targeting young adults with schizophrenia?*
Relatives of individuals with SSD	*What do exercise instructors need to be aware of to meet the needs of relatives in relation to community-based exercise targeting young adults with schizophrenia?*
Young adults with SSD	*What is important for supervised community-based exercise to facilitate long-term physical active behaviour?*

SSD = Schizophrenia spectrum disorders

### Data analysis

A research assistant transcribed the audio recorded material clean verbatim in accordance with conventions described with predefined transcription guideline. MFA validated the transcripts against the audio recordings. NVivo 12 (QRS International, Melbourne, Australia) and Microsoft Excel were employed to facilitate both data management and data analysis. All data and transcription guidelines (in Danish) can be provided upon reasonable request.

Inductive qualitative content analysis, as described by Graneheim and Lundman [30], was applied to analyse the focus group discussions. MFA and KR (female investigator, registered nurse, and full-time PhD student) initially read and reread the transcripts separately before jointly discussing them to obtain a sense of the complete data material. Subsequently, MFA de-contextualised the material, identifying and extracting meaning units before labelling them with descriptive codes (in NVivo). Next MFA compared, abstracted, and sorted codes into categories and subcategories (in Excel), which were discussed with KR and JM (female investigator, psychologist, senior qualitative health researcher, and clinical professor). Categories and subcategories were continuously compared with the original transcripts in an iterative analytic process.

## Results

### Characteristics of informants

Six focus groups were conducted, involving a total of 30 individuals representing five different stakeholder groups in January and February 2022. A total of eight individuals (three young adults with SSD, two relatives of individuals with SSD, two physiotherapists, and one clinical staff at mental health outpatient clinic) who had initially agreed to participate in the focus groups had to cancel their participation due to a positive COVID-19 test. Two focus groups included clinical staff from mental health outpatient services, one of which served as a pilot test of the interview guide. Since the interview guide did not require any revision, the transcript from the pilot focus group was included in the analysis. Table 3 presents the selection criteria, recruitment method and stakeholders’ characteristics.

**Table 3:** Selection, recruitment, and characteristics of stakeholders (*n* = 30)

Stakeholder group	Inclusion criteria	Recruitment	Setting	Participant characteristics
Clinical staff at mental health outpatient clinic	Professional experience from working with young adults with SSD	Personal email to team leader at two outpatient clinics who distributed the invitation all staff at the outpatient clinic.	Conference rooms, outpatient clinics	Two groups: (*N* = 4, *N* = 5) 3 men and 6 women Nurses (*n* = 3), social workers (*n* = 4), occupational therapists (*n* = 2) 2–30 years’ experience working in mental health 1–16 years’ experience working in an outpatient mental health clinic
Young adults with SSD	Lived experience of having a SSD diagnosis (F20–29)* Treated at an outpatient mental health clinic and diagnosed with SSD	Email invitation distributed by peer-board coordinator	Conference room, University of Copenhagen	One group: (*N* = 4) 2 men, 2 women Full-time (*n* = 1), between jobs (*n* = 1), student (*n* = 2) 2–17 years in diagnosed with SSD
Relatives of individuals with SSD	Lived experience of being a relative to an individual with SSD	Email invitation distributed by a manager of an outpatient clinic	Conference room, University College of Northern Denmark, Aalborg One relative participated via a Microsoft Teams due to COVID-19	One group: (*N* = 4) Mothers of individuals with SSD Work in healthcare (nurse, occupational therapist, healthcare worker, physician) Tine since children’s diagnosis 1.5–4 years
Physiotherapists working in mental health centres	Experience working with physical activity and exercise for people SSD	Personal invitation to team leader at a department of physiotherapy, who distributed the invitation to all physiotherapist at the department.	Conference room, mental health services, Capital Region of Denmark	One group: (*N* = 4) 4 Women Educated from 1993–2019, 1.5–25 years’ experience working in the psychiatric field
Exercise instructors with/without experience in coaching people with SSD	At least two years’ experience working as exercise instructor and/or experience with coaching people with SSD	Personal email to exercise instructors coaching people with SSD as part of previous feasibility study** Email invitation distributed by fitness chain	For geographical reasons Microsoft Teams was used	One group: (*N* = 9) 6 men, 3 women Previous experience coaching people with SSD (*n* = 3), no experience (*n* = 6), student (*n* = 4), full-time personal trainer (*n* = 2) 2–8 years’ experience as an exercise instructor

*International Classification of Diseases–Version 10 [31], ** [32], SSD = schizophrenia spectrum disorders

### Findings

Thirty-three unique codes, nine subcategories and three categories were developed. From these findings we derived required elements for an educational programme. Table 4 communicate these required elements and the categories: (i) *awareness and understanding of mental illness*, (ii) *protecting youth identity, and* (iii) *promoting exercise as a shared activity*, with corresponding subcategories and quotes. In the following presentation of the results, lay exercise instructors will be addressed only as *instructors.* Young adults with SSD who are expected to participate in community-based exercise will be addressed only as *participants*. Each subcategory name will be indicated in italics.

**Table 4:** Overview of analysis resulting in required educational elements derived from three categories and nine subcategories with corresponding illustrative quotes

Required educational elements	Categories	Subcategories	Illustrative quotes
a) Basic knowledge in core symptoms of SSD with particular focus on negative symptoms. b) Understanding the impact of antipsychotic medication side effects on the body. c) Analysis of cases demonstrating how symptoms and medical side effects can influence behaviour. d) Reflection on internalized stigma among individuals with SSD.	Awareness and understanding of mental illness	Knowledge kills prejudice	*It would be nice to know something about the illness* [SSD] *before you start as an instructor… it would be nice to confirm or deny some common myths and assumptions regarding schizophrenia.* (Exercise instructor)
		Lack of meaning	*It makes me think about the shame… the feeling of: “Why is this so awkward? Why am I making it awkward? Why can’t I figure out how to be in company with other people?” I have thought a lot about that.* (Young adult with SSD)
		Extra compassion	*The process in getting better is long and thus it may be beneficial to focus on the small things, like “I see you look happier today” or “you could do 20 [repetition of an exercise] and now you can easily do 30”. So, you look at what is small, good, or on the way.* (Clinical staff at mental health outpatient clinic)
a) Reflections on the concept of mental health versus mental illness. b) Practice of respectful and inquisitive communication. c) Knowledge about social inclusion and personal recovery. d) Understanding the concept of proximal zone of development.	Protecting youth identity	Person before the diagnosis	*Two people with the same diagnosis can have totally different symptoms and functional level… they are so much more than just a diagnosis.* (Clinical staff at mental health outpatient clinic)
		Stepping out of the patient role	*So, she [a young adult with SSD] can take a selfie for her friends and tag it with “going to the gym, smiley” helping her maintain an identity similar to her friends.* (Relative of an individual with SSD)
		Good and bad days	*Once, I heard on the radio that you can climb a hill in different ways. You can run, you can walk, and you can crawl…but we all need to get up there.* (Young adult with SSD)
a) Guidelines and procedures for contacting appropriate parties if needed. b) Strategies for combining group facilitation with selective participation. c) reflection on non-fitness related goals in community-based exercise.	Promoting exercise as shared activity	Safe space	*Trust that others [health professionals] have the responsibility to address this, not themselves [instructors]. They are just one part of the journey for the young person to feel better. No further demands should be imposed on the instructors besides doing the best they can and knowing that they are doing something good.* (Relative of an individual with SSD)
		Making exercise meaningful	*Having a playful part at the beginning [of an exercise class] really loosened up the atmosphere…playing around positively affected the dynamic… and ending the class well…having a good experience to go home with.* (Exercise instructor)
		Alone and together	*They can give each other so much. Somebody may contribute with more motivation. Sometimes, you may come up with your own goals, but you may also be inspired by others, and suddenly, you have a common goal.* (Physiotherapists working in mental healthcare)

#### Category 1: Awareness and understanding of mental illness

According to the stakeholders, an educational programme should enable instructors to comprehend and acknowledge the nature of SSD are and how the disorder may affect the life of the person suffering from them. Consequently, equipping instructors’ knowledge and tools for continuous reflection, was considered beneficial in dispelling common myths and stigma associated with mental illness. Based on the assertion that *knowledge kills prejudice*, the stakeholders emphasized the importance of providing instructors with fundamental knowledge about SSD and common side effects of medical treatment, including severe weight gain. However, knowledge should not be provided with the aim of enabling instructors to engage in therapeutic work. Several stakeholders pointed to the balance between knowing too little or too much, as both may lead to an inappropriate focus on the disease. The purpose of the knowledge taught should be to reduce stigma by clarifying that limited motivation, interest, and expression are well-known negative symptoms of SSD and potential side effects of medical treatment, not to be confused with personality traits. Symptoms often fluctuate may manifest as the feeling that life has a *lack of meaning*, leading to periods with self-devaluation and internalized shame among the participants. During these times, participants may be in a vulnerable state of mind, and stressful situations may trigger a fight or flight response. The stakeholders emphasized that instructors may need to show *extra compassion* by acknowledging even the smallest victories and focusing on the effort being made rather than the results being achieved. The instructors’ role is to empower the participants and serve as an external motivator during periods when the participant’s internal motivation might be low.

The required elements for an educational programme derived from this category includes (a) basic knowledge in core symptoms of SSD with particular focus on negative symptoms, (b) understanding the impact of antipsychotic medication side effects on the body, (c) analysis of cases demonstrating how symptoms and medical side effects can influence behaviour, and (d) reflection on internalized stigma among individuals with SSD.

#### Category 2: Protecting youth identity

The stakeholders emphasised that the instructors must meet participants with the same level of curiosity as they would meet anyone else at the gym. They must focus on exploring personal resources to support the protection of youth identity. Thus, an educational programme must inspire instructors to put the *person before the diagnosis* and acknowledge that even though participants may share the same diagnosis, they will all have different difficulties, strengths, preferences, and dreams in life. The stakeholders said that instructors must be encouraged to communicate with curiosity if they observe participants who are experiencing challenges due to their mental illness. Psychotic symptoms, such as hallucinations and delusions may inhibit participants from touching exercise equipment, while negative symptoms, such as apathy and withdrawal from social situations may hinder them from participating in a team workout. The stakeholders fear that instructors may refrain from addressing what is happening to avoid making the situation worse, but this may constitute a disservice to the participants. Instructors should embrace an attitude that shows that they are interested in learning from the participants as experts in their own lives and by building a balanced relationship. Sharing and laughing about everyday problems, thoughts, and experiences may foster an atmosphere where engaging in physical activity with others becomes a natural and integrated part of daily life, serving as a means of *stepping out of the patient role.* To enable participants to be physically active despite potential barriers, stakeholders highlight that an exercise community should be able to embrace participants during *good and bad days*. An educational programme should ideally give instructors the ability to balance the demands of the exercise content with the physical, psychological, and social capacity of the participants. It is crucial to recognize when to take care of special needs and when to encourage participants to out of their comfort zones. However, the stakeholders recommended starting slowly to improve the chance of success, which is why they may benefit from discreetly making note of the energy level of the participants at the beginning of an exercise session.

The required elements for an educational programme derived from this category includes (a) reflections on the concept of mental health versus mental illness, (b) practice of respectful and inquisitive communication, (c) knowledge about social inclusion and personal recovery, and (d) understanding the concept of proximal zone of development.

#### Category 3: Promoting exercise as a shared activity

According to stakeholders, a shared exercise community may become a symbol of the relationship between the participants, their peers, and the instructors which may in turn produce personal growth for everybody involved. Nevertheless, the instructors are responsible for contextual factors that may be crucial to realising the potential of exercising together. As such, community-based exercise should represent a *safe space* for participants that ensures a recognisable and predictable structure and the presence of trusted others. The stakeholders also mentioned that instructors should be adequately prepared for their role as instructors for them to feel comfortable. Instructors will likely be affected if they see signs of self-harm, such as cutting marks and cigarette burns, but their focus should be to support participants in their recovery process and not to solve their difficulties. To facilitate empowerment and sustained engagement in physical activity, the stakeholders emphasized the importance of *making exercise meaningful* for everyone, even though this may be challenging when the exercise is delivered in groups. Aligning expectations may be beneficial in identifying individual goals, and instructors can help support minor improvements based on these goals. For some, this may involve assisting in establishing structure in everyday life; for others, it may involve providing an arena for participants to develop their social skills. Some participants may strive to be strong or fit, while others might seek a distraction from clinical symptoms. However, the stakeholders believe that the overall goal of the exercise should be to make it enjoyable. Community-based exercise should create an environment for being both *alone and together* emphasizing both individual and collective goals. Facilitation of group dynamics is crucial since it fosters a positive, binding community capable of sustaining ongoing participation. Exercising with others, who may face similar challenges due to mental illness may produce an unspoken mutual understanding. As such, instructors should acknowledge community-based exercise as a shared space that participants utilize as a steppingstone towards social inclusion in civil life.

The required elements for an educational programme derived from this category includes (a) guidelines and procedures for contacting appropriate parties if needed, (b) strategies for combining group facilitation with selective participation, and (c) reflection on non-fitness related goals in community-based exercise.

## Discussion

This study aimed to provide perspectives into the required elements of an educational programme for lay exercise instructors engaged in delivering community-based exercise tailored for young adults with SSD. Our objective was to meet the growing demand for sustainable, easily accessible, and engaging community-based initiatives in mental health care [33]. Overall, our findings suggest that educational programmes should allow instructors engage in the highly significant role as guardians of an inclusive culture that embrace an anti-stigma stance by: (a) being aware and understand the difficulties that mental illness may produce; (b) being a protector of youth identity; and (c) being a provider of exercise as a shared activity that symbolises the relationship between participants, their peers, and the instructors. A qualitative review of physical activity among people with enduring mental health difficulties found that exercise instructors were essential in providing a safe and supportive environment while bolstering their sense of competence and self-esteem [34]. Furthermore, our previous qualitative study on the experiences of participants and instructors investigating the feasibility of an exercise programme for people with first-episode psychosis, indicated that lay exercise instructors contributed considerably to a caring yet challenging environment [35]. Instructors may also play an important role in creating a feeling of partaking in normal physical activity with other young adults, helping to reverse the negative stigma associated with mental illness [10].

The stakeholder groups agreed that lay exercise instructors should receive training in basic mental health literacy, which is supported by an international consensus statement [22]. Especially knowledge about negative symptoms as these may often be misinterpreted as inherent personal traits, contributing to stigma against people with mental illness among the general population. Additionally, negative symptoms are highly linked to social functioning and self-efficacy [36], significantly impacting participants’ ability to participate in community-based exercise. Another notable finding across the stakeholders was the importance of the community and that an educational programme should provide the instructors with the skills to facilitate social connectivity among the participants, which is supported by current evidence [10]. Qualitative findings show that the main narratives related to participation in sports and exercise were that the experience of achievement was shared by, and could potentially be shared with, many others in everyday life [37]. Instructors must nevertheless be aware that social interaction is difficult for most people with SSD and may lead to self-isolation [38]. Gym-based group exercise may provide unique flexibility that allows participants to oscillate between socialising and exercising alone [35]. Hence, instructors should be able to tailor exercise content based on physical, social and mental parameters, ensuring that the exercise is meaningful for each participant in the exercise community. Indeed, the motivation for being physically active varies significantly among individuals with SSD. Motivational factors may span from physical outcomes such as weight control and fitness to more psychological and social outcomes like improving mood and meeting new people [12, 39].

The stakeholders pointed to the importance of striking a balance between having too much and too little knowledge. Similarly, the educational content should allow instructors to balance between promoting exercise in a non-patient environment while respecting the limitations and boundaries of SSD. Indeed, based on the assumption that knowledge should serve to promote anti-stigma, education appears to be effective in terms of improving knowledge and fostering positive attitudes, and thereby reducing stigma and discrimination related to mental illness [40]. Furthermore, an educational programme should facilitate ongoing reflections among the instructors to obtain practical knowledge. Qualitative evidence suggests the importance of exchanging experiences and ideas with health professionals and peer instructors when lay instructors supervise physical activity in clinical populations [41]. Also, of importance, exercise instructors may embrace an exercise evangelistic mindset, assuming that exercise participation will invariably contribute to positive personal growth and mental well-being. Therefore, education should clarify that, alongside the potential benefits of exercise and sports, there is also a risk of harm, such as social exclusion or unfavourable comparison to more successful peers [10]. This is especially important considering that initiating community-based exercise in this population may be associated with low self-esteem and the feeling of powerlessness after previously unsuccessful attempts to engage physical activity [7, 9].

Stakeholders touched upon severe weight gain as a medical side effect, and the radical change in body composition that accompanies it may challenge the ability and motivation for physical activity. Interestingly, stakeholders demonstrated limited attention to physical health issues among people with SSD, not focusing on how these issues may serve as both a motivational goal and a potential barrier to physical activity. A systematic review indicate that, exercising to improve general physical health is reported as the most endorsed reason for engaging physical activity among individuals with severe mental illness, while one out of four are citing poor physical health as a barrier to exercise [39]. One explanation may be that stakeholders perceive that addressing physical health issues are within the pre-existing knowledge and competence of lay exercise instructors, making it a less prominent learning objective in an education programme. Nevertheless, it is crucial to ensure to accuracy of this assumption, particularly considering that many individuals with SSD experience lower health literacy (the ability to access, understand and utilise basic health information) compared to the general population [42].

### Methodological considerations

Some limitations and strengths should be considered. A strength is that we recruited individuals from five different stakeholder groups, all highly relevant and with specific knowledge and experience relevant to the objective of the current study. However, due to the COVID-19 pandemic, numerous stakeholders cancelled at the last minute. Consequently, a possible limitation is that only four people from the focus group for young adults SSD and four from the focus group for relatives participated, and they may be characterized by having stronger resources compared to people who were not able or willing to participate. Another limitation of relevance to all groups is that the sampling strategy potentially led to the recruitment of stakeholders with a special interest in, and a predominantly positive attitude towards, exercise. In this regard, relatives were also only represented by mothers working in the healthcare sector, which may challenge the transferability of their perspectives to other relatives, such as fathers and relatives not working in the healthcare sector. During the analysis, the authors felt confident of having achieved or at least closely approached thematic saturation, signifying that additional data would not contribute further to the overall aim of the current study [29]. Using inductive content analysis allowed us to capture the study’s intended objective, which has been underexplored by previous research. Our findings present educational element derived from a comprehensive interpretation of stakeholder perspectives; hence, we acknowledge the influence of our own preconceptions [43]. To enhance credibility, continuous reflections and discussions on preconceptions relating to the study objective were carried out throughout the entire research process [44].

### Implication for practice

The educational elements identified and communicated in the Findings section were adapted and integrated into an educational programme for lay exercise instructors delivering community-based exercise targeting young adults. The educational programme is currently implemented and evaluated as a part of the Vega trial [24] and encompasses (1) a written exercise instructor manual; (2) a one-day educational course; and (3) continuous exchange of experiences between instructors. Appendix  2 provides an overview of the educational programme with headlines on the educational content. The full version of the educational programme in Danish can be provided upon reasonable request.

Exercise interventions within mental health care often aims to improve physical health concerns and clinical symptomatology, thereby prompting exercise instructors to concentrate primarily on prescribing exercise regimes in terms of dosage, intensity, and frequency address physical health exclusively. However, our findings provide learning activities designed to enable exercise instructors to deliver exercise content that fosters youth identity, empowerment and social connectivity, which are core elements in personal recovery [45]. As such, the educational material produced may hold applicability in the training of lay workers engaged in facilitating community-based activities aiming to support the personal recovery journeys of young adults with severe mental illness.

## Conclusion

An educational programme for lay exercise instructors delivering community-based exercise targeting young adults with SSD should emphasize an understanding of the consequence of living with mental illness, strategies to protect youth identity, and methods to promote exercise as a shared activity fostering a sense of community. Indeed, the required elements of such an education programme should empower lay exercise instructors to assume the role as guardians of an inclusive exercise culture. Our results may be transferable to the education of lay workers in mental health care where the aim is to facilitate sustainable, recreational, community-based activities.

### Electronic supplementary material

Below is the link to the electronic supplementary material.

Supplementary Material 1

Supplementary Material 2

## Data Availability

Data and material can be provided upon reasonable request by the corresponding author.
