# Supplementary material for: Required elements for an educational programme for lay exercise instructors in charge of community-based exercise targeting young adults with schizophrenia spectrum disorders – A stakeholder focus group study
Source: BMC Psychiatry. 2024 Mar 26;24:228. doi: 10.1186/s12888-024-05648-9 (PMC10967036; doi:10.1186/s12888-024-05648-9)

Required elements for an educational programme for lay exercise instructors in charge of community-based exercise targeting young adults with schizophrenia spectrum disorders – A stakeholder focus group study

Martin Færch Andersen1,2, Kickan Roed1, Victor Sørensen1, Allan Riis 2,3, Bolette Skjødt Rafn1,4, Bjørn Hylsebeck Ebdrup5,6, Julie Midtgaard1,6

*Correspondence: Martin Færch Andersen, mfan@ucn.dk

1 Centre for Applied Research in Mental Health Care (CARMEN), Mental Health Centre Glostrup, University of Copenhagen, Copenhagen, Denmark

2 Department of Physiotherapy, University College of Northern Denmark, Aalborg, Denmark

Appendix 2 Overview of the educational programme with headlines on the education content (instructor manual and one-day educational course)

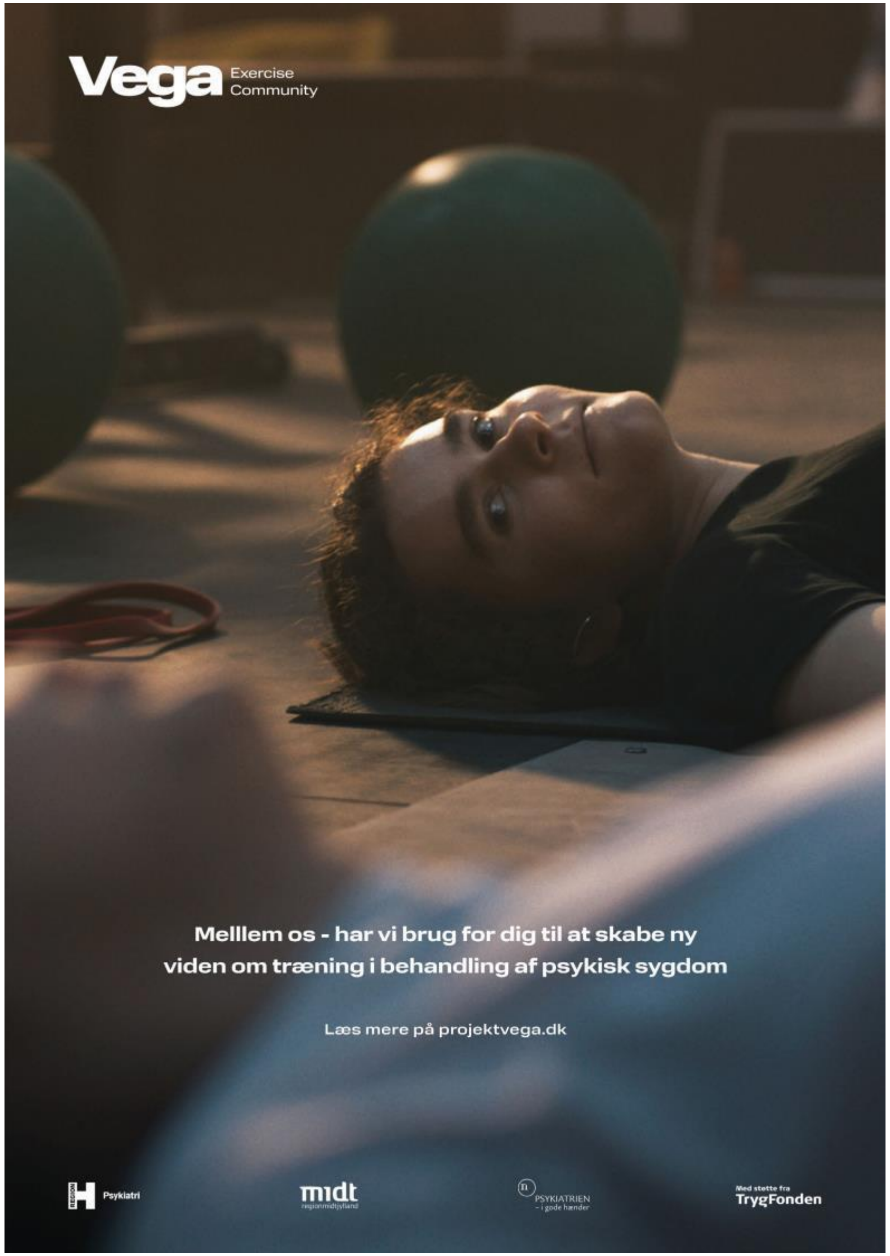

Introduction 3
Project aim 3
Mental health and mental illness 4
Schizophrenia and other psychotic disorders 4
Symptoms 5
Treatment 5
Living with the illness 6
Myths and misunderstandings 6
Mental illness in everyday language 7
Being a Vega exercise instructor 7
Recovery 8
Special attention 10
In case of adverse events.. 11
Vega exercise procotol 13
General structure 13
Adjusting the Vega exercise 13
An exercise session (example) 14
Other working tasks in Vega 16
Exercise self-efficacy meeting 16
Extended support meeting... 18
Administrative registration 18

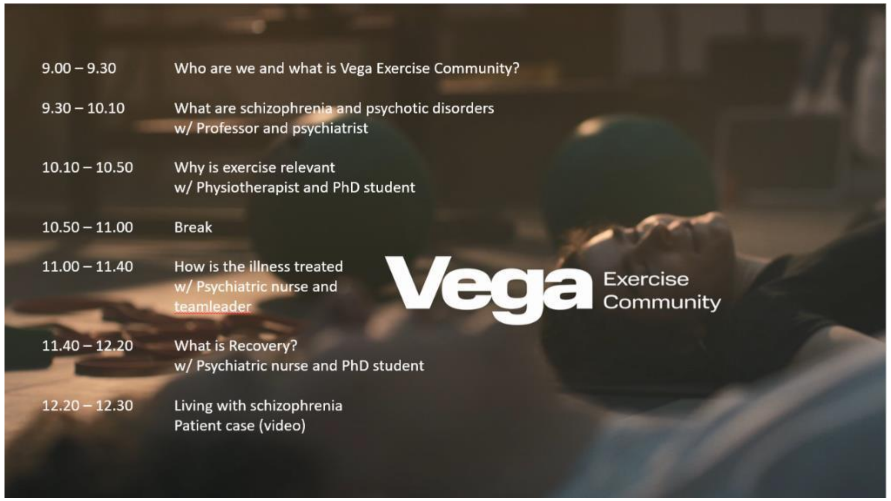

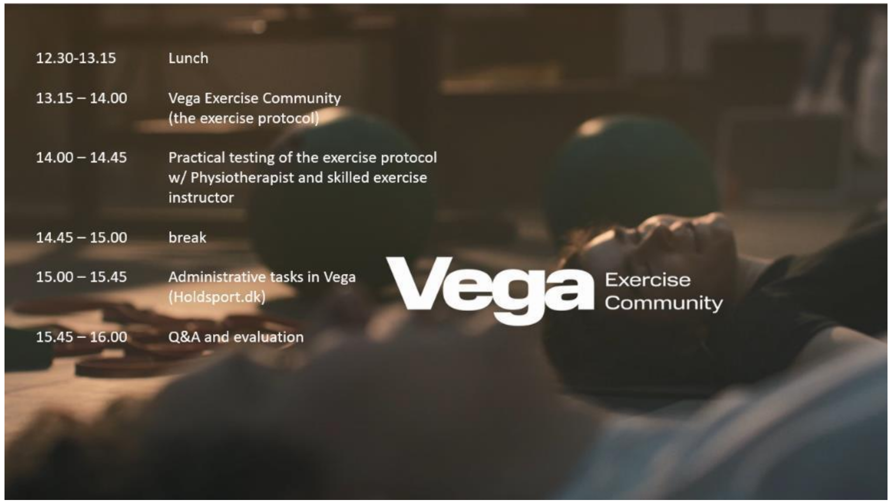

Supplement: Supplementary file 2 — Supplementary Material 2 [file 12888_2024_5648_MOESM2_ESM.pdf]
